# Detection and Phylogenetic Characterization of Canine Distemper Virus (*Morbillivirus canis*) in Wild Foxes and Badgers from Northern Italy in 2021–2026

**DOI:** 10.3390/ani16203217

**Published:** 2026-10-13

**Authors:** Anita Filippi, Stefania Lauzi, Chiara Garbarino, Alessandro Reggiani, Riccardo Rossi, Fabiana Ferrari, Elisa Massella, Andrea Luppi, Annalisa Santi, Ana Moreno, Anna Marelli, Elena Carra

**Affiliations:** 1 Istituto Zooprofilattico Sperimentale Della Lombardia e dell’Emilia-Romagna, 25124 Brescia, Italy; chiaraanna.garbarino@izsler.it (C.G.); alessandro.reggiani@izsler.it (A.R.); elisa.massella@izsler.it (E.M.); andrea.luppi@izsler.it (A.L.); annalisa.santi@izsler.it (A.S.); anamaria.morenomartin@izsler.it (A.M.); anna.marelli@izsler.it (A.M.); elena.carra@izsler.it (E.C.); 2 Dipartimento di Medicina Veterinaria e Scienze Animali, Università Degli Studi di Milano, 26900 Lodi, Italy; stefania.lauzi@unimi.it; 3 Centro Recupero Animali Selvatici di Piacenza—Piacenza Wildlife Rescue Center, 29029 Niviano, Italy; riccardo.rossi@piacenzawildlife.org (R.R.); fabi1981@virgilio.it (F.F.)

**Keywords:** *Morbillivirus*, wildlife, distemper

## Abstract

Canine distemper virus (CDV) is a highly contagious multisystemic pathogen posing severe threats to wild carnivores and domestic animals. To investigate CDV circulation and lineage diversity in Piacenza Province, Northern Italy, wildlife surveillance was conducted under the Regional Wildlife Monitoring Plan between 2021 and June 2026. Forty-three wild carnivores—comprising red foxes (*Vulpes vulpes*), European badgers (*Meles meles*), wolves (*Canis lupus*), stone martens (*Martes foina*), and one jackal (*Canis aureus*)—were evaluated by necropsy, histopathology and molecular assays (RT-qPCR/RT-PCR). Overall, 12 animals (27.9%, 95% CI: 15.3–43.7; six foxes and six badgers) were confirmed to be CDV-positive. No positivity was found before 2024. Positive animals displayed characteristic lesions, including hemorrhagic enteritis, pulmonary hepatization, and footpad hyperkeratosis. Sequencing of the hemagglutinin (H) gene of the four complete H genes obtained revealed that they belonged to clade A of the European/South American 1 lineage, showing the highest nucleotide identity with Alpine CDV strains from Switzerland and Northern Italy. These results confirm the recent circulation of CDV amongst foxes and badgers in Piacenza. Continuous molecular monitoring and integrated cross-border surveillance remain essential for the effective management of transmission at the interface between wildlife and domestic animals.

## Simple Summary

Canine distemper is a highly contagious viral disease that affects domestic dogs and wild animals, including foxes and badgers. In recent years, expanding human activities have increased interactions between wildlife and domestic animals, allowing canine distemper virus (CDV) and other pathogens to spread more easily. To assess the circulation of CDV among wildlife populations, specimens from dead wild animals and live individuals presenting lesions or clinical manifestations suggestive of distemper were examined. Samples were collected within the framework of the Regional Wildlife Monitoring Plan between 2021 and June 2026 in the province of Piacenza, Northern Italy. Out of 43 animals examined, 12 tested positive for CDV, specifically six red foxes and six European badgers. Lesions observed included severe lung, intestinal, or skin damage. Genetic profiling of five strains obtained and sequenced revealed that they are closely linked to those previously identified in Switzerland and in the Lombardy Alpine region. These findings demonstrate the recent circulation of canine distemper amongst the local wildlife of Northern Italy. This work provides information for local health authorities, veterinarians and wildlife conservationists working to protect wildlife, to enable them to understand the local situation and to monitor potential disease outbreaks between wild and domestic animals.

## 1. Introduction

*Morbillivirus canis*, a morbillivirus belonging to the *Paramyxoviridae* family commonly known as canine distemper virus (CDV), is the etiological agent of a highly contagious viral disease affecting both dogs and wildlife species [1,2]. The evolutionary plasticity of the virus has allowed CDV to cross species barriers with extreme efficiency [3,4]. This plasticity is particularly driven by mutations in the hemagglutinin (H) gene, which protein is responsible for binding to host cellular receptors like SLAM (Signaling Lymphocytic Activation Molecule) and Nectin-4. Consequently, its host range has expanded far beyond domestic dogs and wild canids (such as wolves and foxes), impacting families within the order Carnivora, such as Mustelidae, Felidae, Procyonidae, and Ursidae, and non-carnivore taxa [5,6], and further expanding to free-living New World primates [7]. The high genetic diversity of the H gene also supports the distinction of different CDV genetic lineages [8].

Clinically, CDV manifests as a highly contagious, multi-systemic disease whose outcome ranges from subclinical infection to acute mortality, depending on the host species, immune status, and viral lineage virulence [3,9]. CDV can induce a severe, often fatal multisystemic infection affecting the immune, respiratory, gastrointestinal and nervous systems. Typical manifestations include lymphoid depletion, hyperkeratosis, encephalopathy, and interstitial pneumonia (often complicated by secondary bacterial infections). Transmitted via aerosolized oral, nasal, and ocular fluids, this highly contagious virus primarily enters the host through the respiratory tract [2].

The epidemiological impact of CDV on wildlife is deeply tied to the shrinking anthropogenic–wildlife interface. Urbanization, habitat fragmentation, and agricultural expansion have intensified contact between domestic reservoirs and wild populations, facilitating frequent spillover and spillback events [7,10]. While mass vaccination of domestic dogs has historically been the cornerstone of control, recent evidence suggests that CDV is now sustained by complex wildlife “meta-reservoirs” (e.g., raccoons in North America and badgers and foxes in Europe), rendering eradication challenging [3,10]. Even in European ecosystems, continuous genomic surveillance confirms the active circulation of diverse lineages (such as European Wildlife and Arctic-like clades) across wild canids and mustelids, underscoring the unpredictable nature of this pathogen at the domestic–wildlife interface [6,11]. To develop effective conservation policies and mitigation strategies, a comprehensive understanding of how CDV behaves in non-domestic hosts is crucial.

In Italy, as in several European countries, CDV has been detected in various wild species and different regions over the last two decades, raising questions about the actual role of wildlife in the persistence and transmission dynamics of this infection and increasing awareness of the importance of continuous monitoring of this disease [12,13,14,15,16,17].

The province of Piacenza, within the Emilia Romagna region, is among the Italian areas under wildlife health monitoring according to the Regional Wildlife Monitoring Plan that has been active in this region since 2006. This plan aims at identifying pathologies and etiological agents relevant to both the wild and domestic population at an early stage through both active and passive surveillance. In this context, CDV is investigated in the presence of nervous and/or respiratory symptoms reported from the rescue center in animals belonging to the *Carnivora* family and/or in the presence of suspected lesions in these species at post-mortem examination. The first case of CDV was reported in a fox in 2020 in this area (Rossi R. and Lauzi S., personal communication). Furthermore, following reports of distemper in other neighboring provinces [9], the aims of this study were (i) to investigate the circulation of CDV infections in wild susceptible animals in Piacenza Province from 2021, according to sampling strategies defined by the Regional Wildlife Monitoring Plan; (ii) to genetically characterize the different circulating lineages; and (iii) to describe the main clinical and pathological findings in order to allow better monitoring of CDV in wildlife.

## 2. Materials and Methods

### 2.1. Animals and Sampling

**Figure 1:**
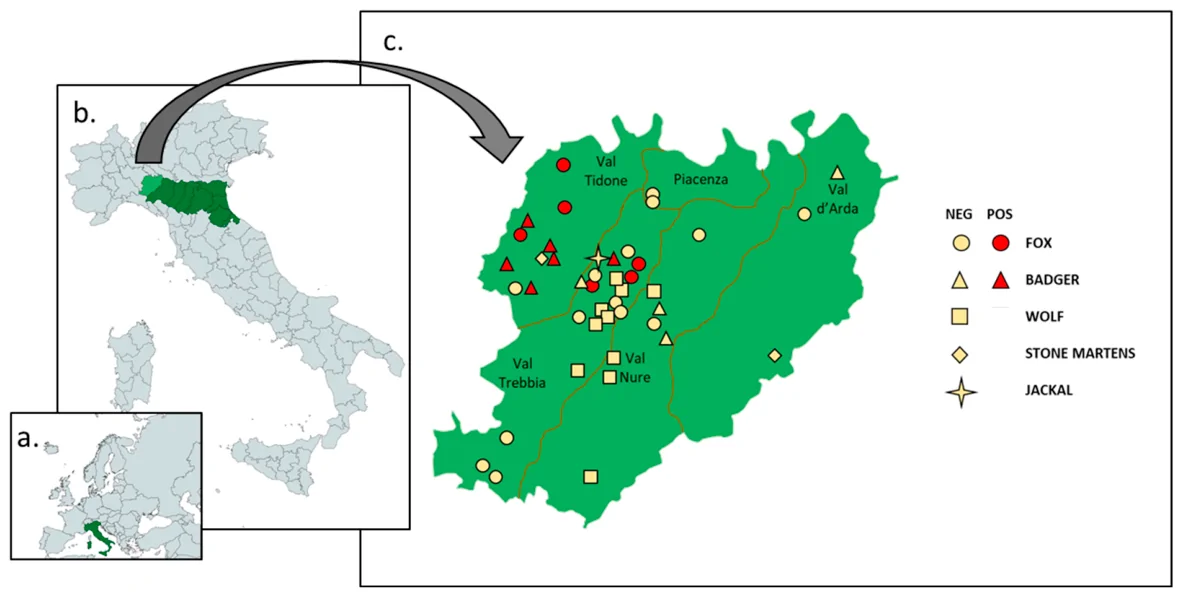
(**a**) Map of Europe, with Italy shown in green. (**b**) Map of Italy, with the Emilia-Romagna region (dark green) and the province of Piacenza (light green). (**c**) Detailed view of the provinces of Piacenza, showing the boundaries of the four valleys (in brown) and the locations of the samples collected and analyzed in this study: the circles represent foxes, the triangles badgers, the squares wolves and the diamonds stone martens; negative samples are shown in yellow, positive samples in red.

The present study included all live wild animals suspected of CDV infection admitted to the Piacenza Wildlife Recovery Center (CRAS) and those found dead in Piacenza Province, which is divided into four main valleys: Val Tidone, Val Trebbia, Val Nure and Val d’Arda (Figure 1). The samples were collected during the period 1 January 2021–30 June 2026.

Notably, animals admitted to the CRAS were submitted to a clinical visit by the veterinarian of the center that recorded the clinical signs. This study therefore implemented a targeted, risk-based sampling approach, carried out on the basis of symptoms and clinical suspicion in animals found alive and on suspected gross lesions observed in carcasses of animals found dead.

In total, samples from 43 animals were analyzed, including 20 red foxes (*Vulpes vulpes*), 10 badgers (*Meles meles*), 10 wolves (*Canis lupus*), two stone martens (*Martes foina*) and one jackal (*Canis aureus*). The majority of animals were found dead (*n* = 34; 79%), while nine live animals were admitted to the CRAS. Seven animals (16.3%) deceased following admission, while two (4.7%) recovered.

Conjunctival swabs (*n* = 6), blood samples (*n* = 5) and/or urine samples (*n* = 1) were taken from nine live animals treated at the CRAS, and these samples were preliminarily analyzed by CDV real-time reverse-transcriptase PCR (RT-qPCR) at the Department of Veterinary Medicine and Animal Sciences of the University of Milan. Animals that were either found dead or that died following the admission to CRAS underwent a post-mortem examination, and the lesions found were recorded at the Piacenza Unit of the Istituto Zooprofilattico della Lombardia e dell’Emilia Romagna (IZSLER) as part of the Regional Wildlife Monitoring Plan. Necropsy examination was performed in all the cases, and gross lesions were recorded. In the presence of a medical history reporting nervous and/or respiratory symptoms and/or intestinal symptoms in animals found alive and in the presence of pneumonia and/or enteritis on post-mortem inspection, central nervous system (CNS), intestinal and respiratory samples were collected from these dead animals. In the case of animals found dead, respiratory, intestinal and/or CNS samples were also collected according to the presence of gross lesions suggestive of CDV. Samples collected from animals were examined in a pool using CDV RT-PCR analysis, and positive amplicons were further sequenced. Forty-one samples of brain, lung, liver and/or skin were taken from dead animals for histopathological examination: the collection of samples was performed according to the condition of the carcasses and the presence of significant pathological lesions.

### 2.2. CDV Detection: RT-qPCR and RT-PCR

Samples collected from live animals and those obtained post-mortem from animals suspected of CDV infection were processed according to two different procedures:

(A) The samples from live animals were sent for a rapid diagnosis (24–48 h) to the Department of Veterinary Medicine and Animal Sciences of the University of Milan. Due to the high transmissibility of the virus, any animal suspected of being CDV-infected was isolated from susceptible animals, and the rapid availability of test results was crucial to enable the prompt implementation of strict biosecurity measures at the CRAS in the event of a positive diagnosis. RNA extraction from samples was performed using a commercial NucleoSpin viral RNA isolation kit (Macherey-Nagel, Bethlehem, PA, USA), following the manufacturer’s instructions. RNA quality control targeting vertebrate the mitochondrially encoded 12S ribosomal RNA locus [18] was performed on samples. Extracted RNA was analyzed by RT-qPCR through the amplification of a conserved 83 bp fragment located within the CDV nucleoprotein (N) gene region, following a previously published protocol with minor modifications [19]. In particular, the RTq-PCR reaction was performed using a QS3 instrument (Applied Biosystems, Waltham, MA, USA), and the RT-qPCR reaction was set up using a commercial kit (TaqMan Fast Virus 1step master mix, Thermo Fisher Scientific, Waltham, MA, USA) in a final volume of 25 μL. After a reverse transcription phase of 5 min at 50 °C and activation for 20 s at 98 °C, 40 cycles with the following conditions were used: 3 s at 95 °C and 1 min at 60 °C. The primers used in this study are those reported in Table 1. As a positive control, RNA extracted from the urine of a dog diagnosed with CDV was used. A blank control (RNase-free water sample) was also included in all RT-qPCR reactions. A sample was considered positive in the presence of an amplification curve and threshold cycles (Ct) < 40, and animals were considered CDV-positive in the presence of at least one positive sample. The RT-qPCR conditioned the management of the case, with the maintenance of the isolation of the positive animal concerned until recovery or death occurred.

(B) The respiratory samples and CNS samples taken post-mortem from animals suspected of having CDV were sent to the Modena Unit of the IZSLER, where they were examined. Viral RNA was extracted from each sample using the QIAsymphony DSP Virus/Pathogen Kit (Qiagen, Hilden, Germany) on a QIAsymphony SP automatic extractor (Qiagen, Hilden, Germany), according to the manufacturer’s instructions, and eluted in 60 µL of elution buffer. Each extract was tested using a One-Step RT-PCR protocol for amplification of the CDV nucleoprotein (NP) gene, using the primers reported in Table 1 [20] and the SuperScript III One-Step RT-PCR System with Platinum Taq DNA Polymerase (Invitrogen, Thermo Fisher Scientific, Waltham, MA, USA), according to the manufacturer’s instructions. Cycling conditions consisted of reverse transcription at 55 °C for 30 min and an initial activation step at 94 °C for 2 min, followed by 40 cycles of denaturation at 94 °C for 15 s, annealing at 58 °C for 30 s, elongation at 68 °C for 30 s, and a final elongation step at 68 °C for 5 min. Positive and negative controls were included in all RT-PCR reactions. The PCR products were separated on a 1.8% agarose gel containing SYBR Green (1X) and visualized on a UV transilluminator, revealing the presence of a CDV amplicon size of 287 bp in the samples and the positive control.

Overall, an animal was considered CDV-positive in the presence of positive post-mortem RT-PCR results.

### 2.3. Sequencing, Phylogeny and Analysis of Complete Gene H

The samples that tested CDV-positive by One-Step RT-PCR were sent to the IZSLER Virology Department in Brescia for sequencing of the complete H gene. Genetic characterization of the samples was based on the complete sequencing of the Morbillivirus H gene, as previously described [9]. Four different primer pairs were used (Table 1), and RT-PCR was conducted using a One-Step RT-PCR kit (Qiagen, Hilden, Germany), according to a previously published protocol [14]. All the RT-PCRs were conducted under the same conditions using the same thermocycler program [21]. Positive and negative controls were included in all RT-PCR reactions.

The PCR products were sequenced using the Sanger method with both the forward and reverse primers used for the RT-PCRs. The resulting sequences were assembled into contigs using the Lasergene Sequencing Analysis Software package, version 12.3.0.57 (DNASTAR, Mad-ison, WI, USA) and compared with sequences in the NCBI BLAST web server (https://blast.ncbi.nlm.nih.gov/Blast.cgi) in 1 August 2026. The sequences were subsequently aligned using ClustalW implemented in MEGA XI, version 11.0.11. Phylogenetic analysis of the complete H gene sequences was performed using IQ-TREE software, galaxy version 3.1.3+galaxy 1. Maximum-likelihood phylogenetic inference was performed using the best-fit nucleotide substitution model identified by ModelFinder (GTR + FO) (available on IQ-TREE software), with 1000 bootstrap replicates [22,23,24], on 163 sequences, with a total length of 1826 nucleotide positions. The resulting tree was midpoint-rooted.

### 2.4. Histopathological Analysis

The histopathological examination was performed on lung, brain, liver and skin samples, depending on the availability and quality of the tissues, from animals suspected of being infected with CDV following post-mortem examination. The tissue samples were fixed in 10% neutral-buffered formalin. Following routine paraffin embedding [25], formalin-fixed tissues were sectioned at 4–5 μm. Sections were stained with hematoxylin and eosin (H&E) and evaluated under a light microscope to systematically identify and record pathological lesions in various organs.

### 2.5. Statistical Analysis

Statistical analysis was performed using RStudio (version 2025.05.1). To ensure rigorous parameter estimation, two-sided 95% confidence intervals (CIs) for binomial proportions were calculated using the binomial exact method.

## 3. Results

### 3.1. CDV Detection

Overall, 12 out of 43 (27.9%, 95% CI: 15.3–43.7) animals were confirmed to be CDV-positive (Figure 1) by One-Step RT-PCR, including six foxes and six badgers. Among the 12 animals that tested positive for CDV post-mortem, intra vitam samples were available from six individuals (three foxes and three badgers), all of which also tested positive for CDV. The characteristics of the animals analyzed are reported in Table 2 and Table S1.

The two animals that recovered from the clinical condition were negative for CDV by RT-qPCR in samples collected intra vitam. It should be noted that the majority of positive cases were found in Val Tidone (8, 66.7%, 95% CI: 34.9–90.1) and the others in the neighboring Val Trebbia (4, 33.3%, 95% CI: 9.9–65.1) (Figure 1).

### 3.2. Clinical and Pathological Findings

Nine out of the 43 animals examined were found alive in 2024–2026 and admitted to the CRAS. The most observed clinical sign in CDV-positive animals found alive was profound depression, which could abruptly progress to periods of excitement. These episodes initially manifested as piloerection and restless movements within the enclosure and could ultimately culminate in convulsive seizures. In addition to these, hyperkeratosis of the foot pads, ocular conjunctival and/or nasal discharge, and intense scratching of the front part of the body, including the shoulders and muzzle, have sometimes been observed in CDV-positive animals found alive.

**Figure 2:**
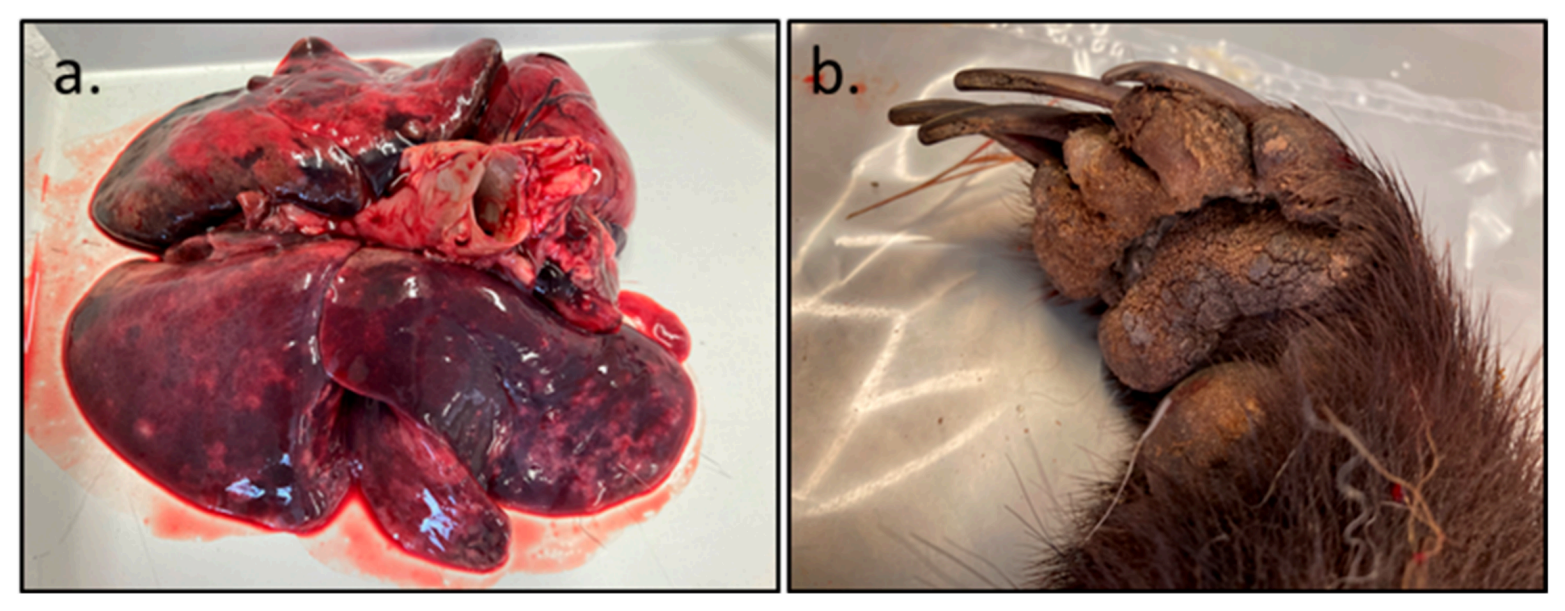
(**a**) Pulmonary hepatization; (**b**) hyperkeratosis of the footpads in one positive badger.

Of the nine animals admitted to the CRAS, seven died and underwent post-mortem examination at the Piacenza Unit of the IZSLER, together with the other 34 animals found dead, for a total of 41 dead animals analyzed. During these examinations, lesions consistent with CDV were identified in CDV-positive animals. In particular, intestinal lesions were found in five cases; three of these were characterized by hemorrhagic enteritis, while the other two showed signs of intestinal congestion. Pneumonia with diffuse hepatization was found in five cases. Furthermore, hyperkeratosis of the footpads was observed in one badger (Figure 2).

The animals that subsequently tested negative for distemper via PCR showed lesions that, even if consistent with distemper infection, cannot be considered specific and pathognomonic. In particular, in nine animals, lesions were found in the lungs, ranging from pulmonary edema and pulmonary congestion to diffuse hepatization. In four other suspected cases, the stomach contents were catarrhal–hemorrhagic, or the animals exhibited pulmonary congestion. Finally, in four animals, areas of alopecia were found on the skin of the limbs.

In CDV-positive animals, tissue samples not necessarily freshly collected but showing apparently adequate morphological preservation and no evident signs of autolysis were selected. Following these criteria, tissues available for histopathological examination were represented by lung (*n* = 4), liver (*n* = 1), intestinal (*n* = 1), skin (*n* = 1) and brain tissue (*n* = 1). Histology revealed a range of lesions affecting mainly the respiratory system, with occasional involvement of the central nervous system, liver, and skin.

**Figure 3:**
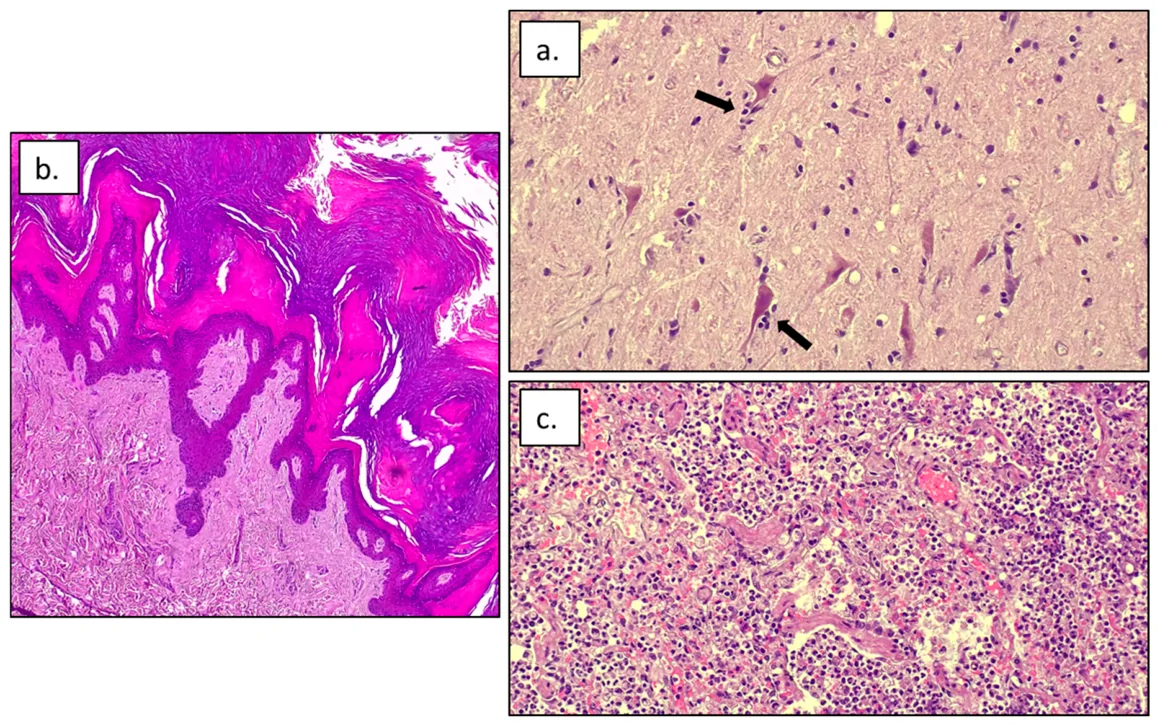
(**a**) Badger positive for CDV. Brain. Satellitosis around neurons can be observed (arrows). Hematoxylin and eosin, magnification 20×. (**b**) Badger positive for CDV. Skin. Foot pads. Widespread severe orthokeratotic hyperkeratosis and hyperplasia of the prickle cell layer of the epidermis (acanthosis). Hematoxylin and eosin, magnification 4×. (**c**) Fox positive for CDV. Lung. The alveolar spaces are filled with large numbers of neutrophils and alveolar macrophages in a case of suppurative bronchopneumonia. Hematoxylin and eosin, magnification 20×.

In particular, in a badger (ID 29), the histological examination showed severe hyperemia, edema and pneumoconiosis in the lungs, while gliosis and satellitosis were observed in the central nervous system (Figure 3a). Moreover, the liver showed signs of autolysis.

In another badger (ID 34), infiltration of macrophages, lymphoplasmacytic cells and neutrophils were observed in the lungs, forming granulomas that were sometimes arranged to surround parasitic elements (eggs, larvae and adult specimens). Severe hyperemia was also observed in both the lungs and the liver.

At the level of the lung parenchyma of a third badger (ID 37), the alveolar spaces were occupied by homogeneous eosinophilic material (oedema), and aggregates of larval nematodes were detected. In the skin, particularly in the plantar pads, four sections were examined in which the prickle cell layer of the epidermis appeared diffusely hyperplastic and an increase in keratohyalin granules (hypergranulosis) was observed (hyperkeratosis) (Figure 3b). Furthermore, severe and widespread thickening of the stratum corneum of the epidermis with anucleated keratin was observed. In the liver, the tissue appeared partially autolytic.

Finally, on examination of the lung sample from a fox (ID 38), numerous multifocal hemorrhages and a multifocal infiltration of neutrophils were observed in the bronchi, bronchioles and alveolar spaces, together with the presence of alveolar macrophages (Figure 3c).

### 3.3. Phylogenetic Analysis

Genetic characterization based on complete sequencing of the H gene was possible for four samples: three from foxes (CDV_Italy_fox_177875_2024_PC_Fo_Italy, CDV_fox_Italy_94580_2026_PC and CDV_fox_Italy_106844_2026_PC) and one from a badger (CDV_Badger_Italy_90334_2026_PC). The complete H gene sequence (1824 nt) was successfully obtained from four of the five samples characterized by sequencing (see Table S1 for AN). For the remaining sample (ID 39: CDV_Badger_Italy_99270_2026_PC), only a partial H gene sequence of 946 nt was generated. Although this partial sequence allowed the identification of the closest matching CDV sequences based on nucleotide identity, it was excluded from the phylogenetic dataset because the complete H gene sequence was not available. As for the other seven strains that were not sequenced, the low RNA quality and low viral load likely did not allow us to obtain informative sequences.

**Figure 4:**
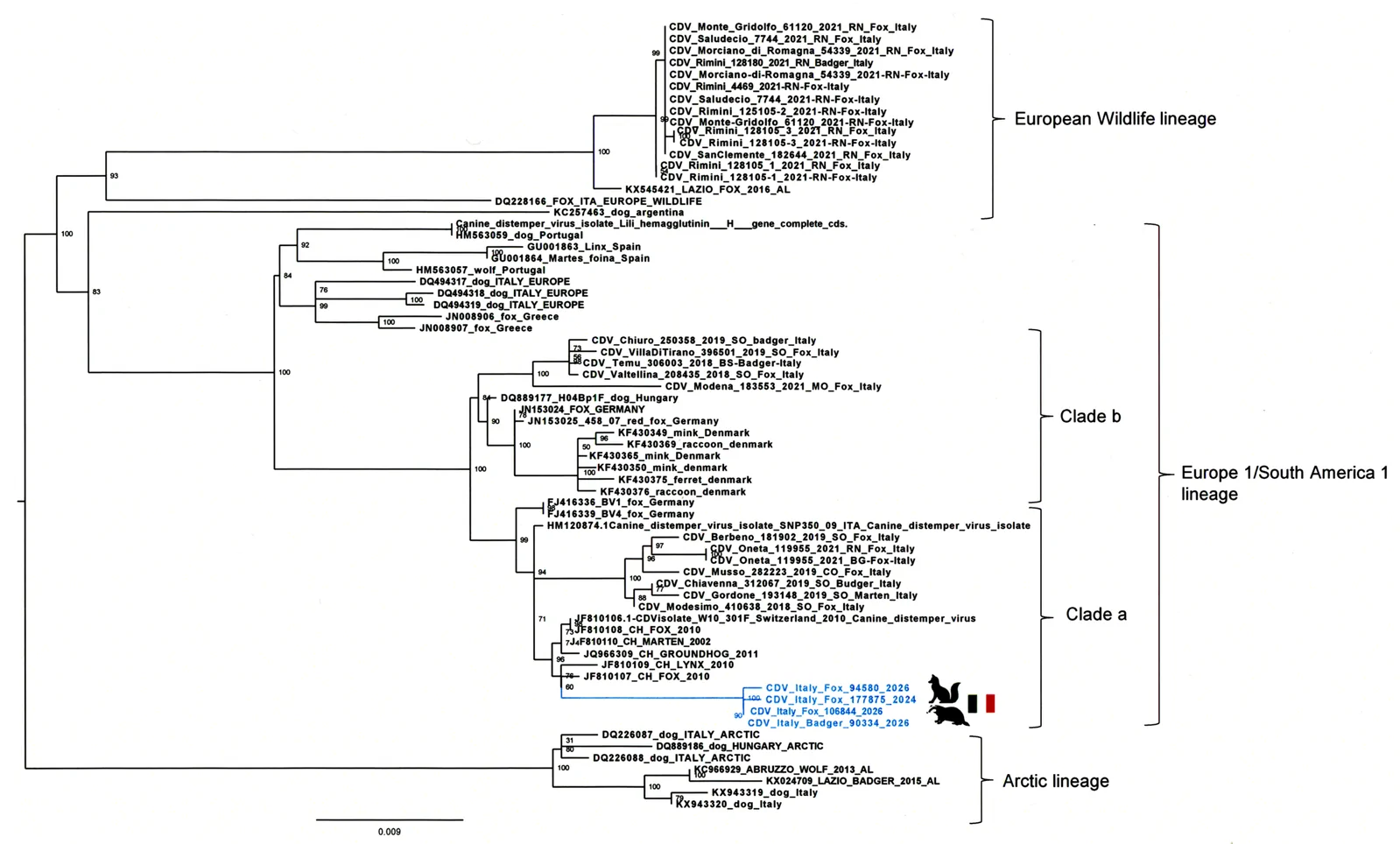
Maximum-likelihood phylogenetic tree based on 68 H gene sequences of 1826 nucleotide positions. All three CDV lineages present in Italy are shown. Italian CDV sequences investigated in this study are highlighted in blue. Bootstrap support values are indicated at the nodes.

Phylogenetic analyses showed that the four whole H-CDV sequences obtained in this study belonged to the European/South America 1 lineage (Figure 4). Within this lineage, two distinct clades have previously been identified in Italy and were originally associated with two epidemic waves occurring during 2006–2009 and 2011–2018, respectively [26]. More recently, the distribution of Italian CDV sequences within these two clades has been shown to be associated with their geographic origin rather than with their temporal distribution [9].

BLAST analysis showed that, for the five sequences (the four full sequences + the partial sequence previously excluded, i.e., CDV_Badger_Italy_99270_2026_PC), except sample 38, the highest nucleotide identity was observed with a CDV strain isolated from a red fox in Switzerland [27]. In contrast, sample 38 (CDV_fox_Italy_106844_2026_PC) showed the highest nucleotide identity with an Italian CDV strain isolated from a red fox in the Italian Alps [28]. The nucleotide identity percentages for each sequence are reported in Table 3. Both reference strains are closely related and belong to the same clade (clade A) of the European/South America 1 lineage. They were isolated from red foxes during the 2009–2010 CDV outbreaks in Switzerland and in Stelvio National Park, Italy, respectively.

Complete sequences obtained in this study were deposited in GenBank under accession numbers PZ871211, PZ871212, PZ871213, and PZ871214.

## 4. Discussion

Following the first detection of canine distemper virus in the province of Piacenza, Emilia-Romagna Region, Northern Italy, in 2020, the present study investigated the circulation of the virus in wild carnivores between 2021 and June 2026 within the framework of the Regional Wildlife Monitoring Plan. The surveyed species included red foxes, European badgers, stone martens, wolves, and a single golden jackal.

Previous phylogenetic studies on CDV strains detected in wild carnivores from the Emilia-Romagna region demonstrated the circulation of two distinct lineages characterized by clear geographic segregation [9,29]. Sequences from the Romagna area clustered within the European Wildlife lineage, whereas those from Emilia belonged to the European/South America 1 lineage. Within the latter, a sequence previously reported from Modena Province clustered in clade B alongside sequences from the northeastern Italian Alps, suggesting an epidemiological link with strains circulating in the Balkan region [9].

Animals infected with CDV displayed a broad range of manifestations, including non-specific symptoms such as fever, lethargy, and anorexia, as well as respiratory, gastrointestinal, and neurological involvement [30,31]. Although neurological signs are frequently associated with CDV, they are not pathognomonic and can occur in other infectious or non-infectious conditions [32]. Indeed, the neurological symptoms observed in positive animals found alive are often similar to those seen in cases of poisoning, particularly by acetylcholinesterase inhibitors or pyrethroids. Therefore, clinical findings must always be interpreted alongside laboratory confirmation.

In our study, 12 animals tested positive (six foxes and six badgers), exhibiting typical distemper signs such as hemorrhagic enteritis, pneumonia with diffuse hepatization, and footpad hyperkeratosis—the latter being a highly characteristic cutaneous indicator of infection. However, gross lesions suggestive of CDV are not specific and require further diagnostic confirmation for CDV diagnosis as these lesions were also found in CDV-negative animals, and other causes, such as parasites, bacteria or poisoning cannot be ruled out. The pathological findings detected, even if CDV-compatible, cannot be considered CDV-specific lesions (such as characteristic viral inclusion bodies, syncytia and demyelination). The evaluation and interpretation of the pathological findings is also limited by the fact that fresh tissues were not always available for histopathological examinations and that immunohistochemistry or in situ hybridization tests were not performed. For this reason, the histological lesions described cannot be conclusively attributed to CDV.

Notably, most positive cases were concentrated in Val Tidone (8/12, 66.7%, 95% CI: 34.9–90.1) and the neighboring Val Trebbia (4/12, 33.3%, 95% CI: 9.9–65.1). The overall detection across species and the spatial clustering suggests the possible presence of persistent circulation of CDV in the local area, which requires further systematic monitoring. However, these numbers may underestimate the true extent of CDV circulation. As previous studies suggest, CDV can be well adapted to wild carnivores, often circulating in a subclinical or mildly symptomatic form [9]. Consequently, infected individuals lacking a clear clinical history or pathognomonic lesions may go undetected when surveillance relies solely on clinical or pathological observation. Integrating molecular diagnostic approaches for all monitored wildlife—including asymptomatic animals—is therefore crucial to enable early detection and clarify the actual viral distribution.

Furthermore, previous studies have reported that CDV poses a serious health threat due to its ability to circulate bidirectionally between wild carnivores and domestic dogs, particularly in areas where these populations overlap. In 2012, Origgi et al. [33] described how a sequence isolated from a dog was closely related to sequences obtained from the 2009–2010 Swiss outbreak, which were isolated from European badgers, red foxes, and stone martens. Other recent studies confirm the concomitant circulation of CDV isolates at the wildlife–pet interface, highlighting how urban and peri-urban environments facilitate spillover events between species [3,10,28,34]. Further studies based on molecular genotyping to trace the dynamics of cross-transmission are also needed to investigate the risk of CDV spillover from wildlife to pets in Piacenza Province, with the purpose of implementing the integrated surveillance and control strategies involving both veterinary and wildlife management interventions [10].

This study had several limitations: firstly, it was based on a targeted/risk-based surveillance sample, which was not random and was therefore not representative of the entire wild population; furthermore, the small sample size, combined with the varying numbers of animals across years, species and valleys, means that the observed positive results cannot be interpreted as indicating prevalence within the population. Moreover, the spatial concentration could be due to potential biases linked to the recovery of carcasses or geographical sampling; these limitations make it impossible to infer prevalence, the direction of transmission or the maintenance of endemicity. Moreover, a stratified analysis by year, sample type, detection method or animal source was not carried out. Nevertheless, an apparent increase in CDV positivity was observed from 2024 to 2026, and even if this trend cannot be interpreted as a true epidemiological rise, it highlights the need for continuous monitoring with larger sample sizes to distinguish genuine epidemiological changes from stochastic variations in prevalence. In addition, it cannot be ruled out that the absence of positive results in 2021–2023 and the increase in positive results in 2024–2026 was due to changes in testing strategy, given that RT-qPCR was introduced for samples from animals found alive starting from 2024, when live animals with clinical signs suggestive of CDV were observed. In any case, the detection of CDV in multiple wildlife species, together with the apparent increase in positivity observed in recent years, highlights the importance of maintaining systematic and long-term surveillance in wildlife populations. Such surveillance can also provide valuable information for the conservation and management of vulnerable or threatened species, whilst contributing to the assessment of the potential risk posed by CDV strains present in wildlife and domestic animal populations.

A recent study by Gao et al. identifies some climatic factors as being responsible for the increased risk of direct transmission in viral outbreaks in carnivores [35]. Although this study was not designed to evaluate climatic variables, climate change represents a relevant factor for future epidemiological studies, especially regarding its impact on host density, spatial distribution, and interspecific interactions.

Phylogenetic analysis revealed that the four H gene sequences obtained in this study clustered within clade A, alongside sequences previously detected in Switzerland and Valchiavenna (a valley situated in Northern Italy, bordering Switzerland) [21]. Although not all positive samples were sequenced and the four characterized viruses may not represent genetic features of all circulating viruses, these findings further confirm the geographic structuring of CDV genetic diversity within Northern Italy. They indicate that strains circulating in the Piacenza area are genetically more closely related to Swiss and Alpine border strains than to those previously reported from other parts of Emilia-Romagna, suggesting a potential cross-border transmission of the virus. Further studies are needed to investigate the origin of the virus. Nevertheless, this finding underlines the importance of accounting for inter-regional and cross-border wildlife movements when investigating CDV epidemiology.

## 5. Conclusions

Overall, the findings confirm the recent circulation of canine distemper virus (CDV) among wild carnivores in the province of Piacenza and provide further evidence of its genetic diversity and geographic structuring in Northern Italy. Continued monitoring integrating clinical, pathological, molecular, phylogenetic, and environmental data will be essential to better characterize the temporal and spatial dynamics of CDV and its genetic features with a view to conducting future epidemiological studies.

## Figures and Tables

**Table 1:** Primers used in this study for CDV RNA detection and genetic characterization.

Target	Primer	Sequence 5′-3′	Length (bp)	Reference
N gene	CDV-F	AGCTAGTTTCATCTTAACTATCAAATT	83	[19]
	CDV-R	TTAACTCTCCAGAAAACTCATGC		
	CDV-Pb	FAM-ACCCAAGAGCCGGATACATAGTTTCAATGC-TAMRA		
NP gene	p5- F	ACAGGATTGCTGAGGACCTAT	287	[20]
	p6-R	CAAGATAACCATGTACGGTGC		
H gene 1st PCR	72F	TACTCTGGTCACACGTCTTA	726	[14]
	798R	TAGCTCCACTGCATCTGTAT		
H gene 2nd PCR	472F	CCGTACATCACCAAGTCATA	700	[14]
	1172R	TAGAACACCACCTTGTGAAC		
H gene 3rd PCR	1131F	GTAGATGAGAGCACCGTATT	640	[14]
	1771R	TGTGTAGGCAACACCACTAA		
H gene 4th PCR	1629F	GGAGACCAGTTCACTGTAAT	592	[14]
	2221R	GATGGACCTCAGGATATAGA		

**Table 2:** Animals analyzed in this study and CDV results.

Category	Variable	N. CDV-Positive/N. Tested (%; 95%CI)
Species	Red fox	6/20 (30; 11.9–54.3)
	Badger	6/10 (60; 26.2–87.8)
	Wolf	0/10 (0–30.9)
	Stone marten	0/2 (0–84.2)
	Jackal	0/1 (0–97.5)
Age *	Young	4/7 (57.1; 18.4–90.1)
	Adult	8/31 (25.8; 11.8–44.6)
Sex **	Female	4/15 (26.7; 7.8–55.1)
	Male	6/22 (27.3; 10.7–50.2)
Location	Piacenza District	0/2 (0–84.2)
	Val Tidone	8/10 (80; 44.4–97.5)
	Val Trebbia	4/20 (20; 5.7–43.7)
	Val Nure	0/8 (0–36.9)
	Val d’Arda	0/3 (0–70.8)
Year	2021	0/2 (0–84.2)
	2022	0/5 (0–52.2)
	2023	0/9 (0–33.6)
	2024	2/10 (20; 2.5–55.6)
	2025	5/9 (55.6; 21.2–86.3)
	2026	5/8 (62.5; 24.5–91.5)
Total		12/43 (27.9; 15.3–43.7)

* Age data are available for 38 animals and not available for 5 animals; ** sex data are available for 37 animals and not available for 6 animals.

**Table 3:** Percentage identity calculated using the NCBI BLAST web service. The highest percentage identity value is indicated in bold. Percentage identities are reported only for two reference strains: the closest strain (the sole representative of the group of strains sharing a similar geographical origin) and the closest representative among the Italian strains isolated in the Alps.

ID	Sequences	JF810106.1_W10/301F_2010 Switzerland	HM120874.1_SNP350/09/ITA Italy
40	CDV_fox_Italy_106844_2026_PC	**98.79%**	98.68%
19	CDV_Italy_fox_177875_2024_PC_Fo_Italy	**98.74%**	98.63%
37	CDV_Badger_Italy_90334_2026_PC	**98.85%**	98.68%
38	CDV_fox_Italy_94580_2026_PC	98.70%	**98.81%**
39	CDV_Badger_Italy_99270_2026_PC *	**98.73%**	98.31%

* Partial sequence (946 nt).

## Data Availability

Sequences obtained in this study were deposited in GenBank under accession numbers PZ871211 (CDV_fox_Italy_106844_2026_PC), PZ871212 (CDV_Italy_fox_177875_2024_PC_Fo_Italy), PZ871213 (CDV_Badger_Italy_90334_2026_PC), and PZ871214 (CDV_fox_Italy_94580_2026_PC).

## Supplementary Materials

The following supporting information can be downloaded at https://www.mdpi.com/article/10.3390/ani16203217/s1, Table S1: Characteristics of the animals analyzed.

## Abbreviations

The following abbreviations are used in this manuscript:

CDV	Canine Distemper Virus
CRAS	Centro di Recupero Animali Selvatici
IZSLER	Istituto Zooprofilattico Sperimentale della Lombardia e dell’Emilia Romagna

## References

[1] Karki M., Rajak K.K., Singh R.P. (2022). Canine morbillivirus (CDV): A review on current status, emergence and the diagnostics. Virus Dis..

[2] Wipf A., Perez-Cutillas P., Ortega N., Huertas-López A., Martínez-Carrasco C., Candela M.G. (2025). Geographical Distribution of Carnivore Hosts and Genotypes of Canine Distemper Virus (CDV) Worldwide: A Scoping Review and Spatial Meta-Analysis. Transbound. Emerg. Dis..

[3] Wilkes R.P. (2022). Canine Distemper Virus in Endangered Species: Species Jump, Clinical Variations, and Vaccination. Pathogens.

[4] Wang H., Guo H., Hein V.G., Xu Y., Yu S., Wang X. (2023). The evolutionary dynamics history of canine distemper virus through analysis of the hemagglutinin gene during 1930–2020. Eur. J. Wildl. Res..

[5] Rendon-Marin S., Martinez-Gutierrez M., Suarez J.A., Ruiz-Saenz J. (2020). Canine Distemper Virus (CDV) Transit Through the Americas: Need to Assess the Impact of CDV Infection on Species Conservation. Front. Microbiol..

[6] Alfano F., Lucibelli M.G., D’Alessio N., Auriemma C., Rea S., Sgroi G., Lucente M.S., Pellegrini F., Diakoudi G., De Carlo E. (2025). Detection of canine distemper virus in wildlife in Italy (2022–2024). Front. Vet. Sci..

[7] Wilson T.M., de Sousa D.E.R., Macêdo I.L., Machado M., Costa G.R.d.T., Fair P., Ritter J.M., Bullock H.A., DeLeon-Carnes M., Lee E. (2025). Emergence of a Novel Canine Distemper Virus Variant in Urbanized Free-Ranging Marmosets (*Callithrix penicillata*). Transbound. Emerg. Dis..

[8] Duque-Valencia J., Forero-Muñoz N.R., Díaz F.J., Martins E., Barato P., Ruiz-Saenz J. (2019). Phylogenetic evidence of the intercontinental circulation of a Canine distemper virus lineage in the Americas. Sci. Rep..

[9] Trogu T., Castelli A., Canziani S., Tolini C., Carrera M., Sozzi E., Lelli D., Tosi G., Fiorentini L., Di Donato A. (2022). Detection and Molecular Characterization of Canine Distemper Virus in Wildlife from Northern Italy. Pathogens.

[10] Kapil S., Yeary T.J. (2011). Canine distemper spillover in domestic dogs from urban wildlife. Vet. Clin. N. Am. Small Anim. Pract..

[11] Lanszki Z., Lanszki J., Tóth G.E., Cserkész T., Csorba G., Görföl T., Csathó A.I., Jakab F., Kemenesi G. (2022). Detection and sequence analysis of Canine morbillivirus in multiple species of the Mustelidae family. BMC Vet. Res..

[12] Monne I., Fusaro A., Valastro V., Citterio C., Pozza M.D., Obber F., Trevisiol K., Cova M., De Benedictis P., Bregoli M. (2011). A distinct CDV genotype causing a major epidemic in Alpine wildlife. Vet. Microbiol..

[13] Müller A., Silva E., Santos N., Thompson G. (2011). Domestic dog origin of canine distemper virus in free-ranging wolves in Portugal as revealed by hemagglutinin gene characterization. J. Wildl. Dis..

[14] Sekulin K., Hafner-Marx A., Kolodziejek J., Janik D., Schmidt P., Nowotny N. (2011). Emergence of canine distemper in Bavarian wildlife associated with a specific amino acid exchange in the haemagglutinin protein. Vet. J..

[15] Di Francesco C.E., Smoglica C., Angelucci S. (2020). Infectious Diseases and Wildlife Conservation Medicine: The Case of the Canine Distemper in European Wolf Population. Animals.

[16] Ferrara G., Brocherel G., Falorni B., Gori R., Pagnini U., Montagnaro S. (2023). A retrospective serosurvey of selected pathogens in red foxes (*Vulpes vulpes*) in the Tuscany region, Italy. Acta Vet. Scand..

[17] Coradduzza E., Fiori M.S., Pintus D., Ferretti L., Ledda A., Chessa G.S., Rocchigiani A.M., Lostia G., Rossi R., Cancedda M.G. (2024). Canine Distemper Virus in Sardinia, Italy: Detection and Phylogenetic Analysis in Foxes. Animals.

[18] Kitano T., Umetsu K., Tian W., Osawa M. (2007). Two universal primer sets for species identification among vertebrates. Int. J. Legal Med..

[19] Elia G., Decaro N., Martella V., Cirone F., Lucente M.S., Lorusso E., Di Trani L., Buonavoglia C. (2006). Detection of canine distemper virus in dogs by real-time RT-PCR. J. Virol. Methods.

[20] Frisk A.L., König M., Moritz A., Baumgärtner W. (1999). Detection of canine distemper virus nucleoprotein RNA by reverse transcription-PCR using serum, whole blood, and cerebrospinal fluid from dogs with distemper. J. Clin. Microbiol..

[21] Trogu T., Canziani S., Salvato S., Bianchi A., Bertoletti I., Gibelli L.R., Alborali G.L., Barbieri I., Gaffuri A., Sala G. (2021). Canine Distemper Outbreaks in Wild Carnivores in Northern Italy. Viruses.

[22] Nguyen L.T., Schmidt H.A., von Haeseler A., Minh B.Q. (2015). IQ-TREE: A fast and effective stochastic algorithm for estimating maximum-likelihood phylogenies. Mol. Biol. Evol..

[23] Hoang D.T., Chernomor O., von Haeseler A., Minh B.Q., Vinh L.S. (2018). UFBoot2: Improving the Ultrafast Bootstrap Approximation. Mol. Biol. Evol..

[24] Wong T.K.F., Ly-Trong N., Ren H., Demotte P., Baños H., Roger A.J., Susko E., Bielow C., De Maio N., Goldman N. (2026). IQ-TREE 3: Phylogenomic inference software using complex evolutionary models. Mol. Biol. Evol..

[25] Bancroft J.D., Layton C. (2018). The hematoxylins and eosin. Bancroft’s Theory and Practice of Histological Techniques.

[26] Bianco A., Zecchin B., Fusaro A., Schivo A., Ormelli S., Bregoli M., Citterio C.V., Obber F., Dellamaria D., Trevisiol K. (2020). Two waves of canine distemper virus showing different spatio-temporal dynamics in Alpine wildlife (2006–2018). Infect. Genet. Evol..

[27] Sattler U., Khosravi M., Avila M., Pilo P., Langedijk J.P., Ader-Ebert N., Alves L.A., Plattet P., Origgi F.C. (2014). Identification of amino acid substitutions with compensational effects in the attachment protein of canine distemper virus. J. Virol..

[28] Martella V., Bianchi A., Bertoletti I., Pedrotti L., Gugiatti A., Catella A., Cordioli P., Lucente M.S., Elia G., Buonavoglia C. (2010). Canine distemper epizootic among red foxes, Italy. Emerg. Infect. Dis..

[29] Balboni A., Savini F., Scagliarini A., Berti E., Naldi M., Urbani L., Fontana M.C., Carra E., Gibelli L.R.M., Gobbo F. (2021). Natural distemper infection in stone martens (*Martes foina*): From infection to neutralizing antibodies. Res. Vet. Sci..

[30] Loots A.K., Du Plessis M., Dalton D.L., Mitchell E., Venter E.H. (2017). Genome Sequences of Three Vaccine Strains and Two Wild-Type Canine Distemper Virus Strains from a Recent Disease Outbreak in South Africa. Genome Announc..

[31] Freire H.L., Iara Í.H.N., Ribeiro L.S.R., Gonçalves P.A.O., Matta D.H., Torres B.B.J. (2025). Neurological Manifestation of Canine Distemper Virus: Increased Risk in Young Shih Tzu and Lhasa Apso with Seasonal Prevalence in Autumn. Viruses.

[32] Martella V., Elia G., Buonavoglia C. (2008). Canine distemper virus. Vet. Clin. N. Am. Small Anim. Pract..

[33] Origgi F.C., Plattet P., Sattler U., Robert N., Casaubon J., Mavrot F., Pewsner M., Wu N., Giovannini S., Oevermann A. (2012). Emergence of canine distemper virus strains with modified molecular signature and enhanced neuronal tropism leading to high mortality in wild carnivores. Vet. Pathol..

[34] Rios-Usuga C., Ortiz-Pineda M.C., Aguirre-Catolico S.D., Quiroz V.H., Ruiz-Saenz J. (2025). Concurrent Circulation of Canine Distemper Virus (South America-4 Lineage) at the Wild-Domestic Canid Interface in Aburrá Valley, Colombia. Viruses.

[35] Gao H., Li Y., Zhang C., Wang H., Nie Y. (2025). Effects of climate, virus, and host characteristics on the seroprevalence of pathogenic viruses in terrestrial mammals. Conserv. Biol..

