# Supplementary material for: Detection and Phylogenetic Characterization of Canine Distemper Virus (Morbillivirus canis) in Wild Foxes and Badgers from Northern Italy in 2021–2026
Source: Animals (Basel). 2026 Oct 13;16(20):3217. doi: 10.3390/ani16203217 (PMC13700601; doi:10.3390/ani16203217)
Supplement: Supplementary file 1 [file animals-16-03217-s001.zip › animals-4554234-supplementary.pdf]

Table S1. Characteristics of the animals analysed.

ID	Animal found alive/dead	Specie	Sex (M/F)	Adult/Young (A/Y)	Collection Date	Collection Point	Alive animal sample (RT-qPCR, Ct)			Dead animal pooled tissue sample	RT-PCR	Overall CDV Results*	Sequences ID	Accession number	Outcome
							Eye swab	blood	urine						
**1**	dead	fox	-	-	13/01/2021	Val Trebbia	-	-	-	tissue	NEG	NEG			dead
**2**	dead	jackal	M	SUB A	05/07/2021	Val Trebbia	-	-	-	tissue	NEG	NEG			dead
**3**	dead	wolf	M	SUB A	10/11/2022	Val Trebbia	-	-	-	tissue	NEG	NEG			dead
**4**	dead	wolf	F	SUB A	14/11/2022	Val Trebbia	-	-	-	tissue	NEG	NEG			dead
**5**	dead	wolf	F	SUB A	18/11/2022	Val Trebbia	-	-	-	tissue	NEG	NEG			dead
**6**	dead	wolf	M	SUB A	21/11/2022	Val Trebbia	-	-	-	tissue	NEG	NEG			dead
**7**	dead	wolf	F	SUB A	21/11/2022	Val Trebbia	-	-	-	tissue	NEG	NEG			dead
**8**	dead	wolf	M	SUB A	14/02/2023	Val Nure	-	-	-	tissue	NEG	NEG			dead
**9**	dead	wolf	M	SUB A	16/02/2023	Val Nure	-	-	-	tissue	NEG	NEG			dead
**10**	dead	fox	M	A	17/02/2023	Piacenza	-	-	-	tissue	NEG	NEG			dead
**11**	dead	fox	-	-	17/02/2023	Val d'Arda	-	-	-	tissue	NEG	NEG			dead
**12**	dead	wolf	M	SUB A	20/02/2023	Val Nure	-	-	-	tissue	NEG	NEG			dead
**13**	dead	fox	-	-	21/02/2023	Val Trebbia	-	-	-	tissue	NEG	NEG			dead
**14**	dead	fox	F	A	23/02/2023	Val Trebbia	-	-	-	tissue	NEG	NEG			dead
**15**	dead	fox	-	-	05/06/2023	Piacenza	-	-	-	tissue	NEG	NEG			dead
**16**	dead	fox	M	Y	20/07/2023	Val Trebbia	-	-	-	tissue	NEG	NEG			dead
**17**	dead	fox	M	A	04/01/2024	Val Nure	-	-	-	tissue	NEG	NEG			dead
**18**	dead	fox	M	SUB A	04/01/2024	Val Tidone	-	-	-	tissue	NEG	NEG			dead
**19**	alive	fox	F	SUB A	07/06/2024	Val Trebbia	POS, 18,6	-	-	tissue	POS	POS	CDV_Italy_fox_177875_2024_PC_Fo_Italy	PZ871212	dead
**20**	dead	fox	M	Y	02/07/2024	Val Trebbia	-	-	-	tissue	POS	POS			dead
**21**	dead	stone marten	M	A	09/07/2024	Val Tidone	-	-	-	tissue	NEG	NEG			dead
**22**	dead	fox	F	A	28/07/2024	Val Trebbia	-	-	-	swab	NEG	NEG			dead
**23**	dead	fox	F	A	09/09/2024	Val Trebbia	-	-	-	tissue	NEG	NEG			dead
**24**	alive	badger	F	A	05/11/2024	Val Nure	-	POS,35,1	POS,28,7	tissue	NEG	NEG			dead
**25**	dead	wolf	F	A	27/12/2024	Val Trebbia	-	-	-	tissue	NEG	NEG			dead
**26**	dead	wolf	M	-	30/12/2024	Val Nure	-	-	-	tissue	NEG	NEG			dead
**27**	alive	badger	F	A	07/01/2025	Val d'Arda	-	neg	-			NEG			recovered
**28**	dead	fox	M	Y	27/02/2025	Val Nure	-	-	-	tissue	NEG	NEG			dead
**29**	alive	badger	M	Y	30/05/2025	Val Trebbia	POS, 22,4	-	-	tissue	POS	POS			dead
**30**	alive	stone marten	F	Y	06/06/2025	Val d'Arda	neg	-	-			NEG			recovered
**31**	dead	badger	M	A	13/06/2025	Val Nure	-	-	-	tissue	NEG	NEG			dead
**32**	dead	badger	M	SUB A	14/07/2025	Val Tidone	-	-	-	tissue	POS	POS			dead
**33**	alive	badger	-	A	05/09/2025	Val Tidone	-	POS,32,8	-	tissue	POS	POS			dead
**34**	dead	badger	F	SUB A	23/09/2025	Val Tidone	-	-	-	tissue	POS	POS			dead
**35**	alive	fox	M	A	18/12/2025	Val Tidone	POS,20,5	POS,24,6	-	tissue	POS	POS			dead
**36**	dead	fox	F	A	03/02/2026	Val Trebbia	-	-	-	tissue	NEG	NEG			dead
**37**	alive	badger	M	A	04/05/2026	Val Tidone	POS, 19,1	POS,37,6	-	tissue	POS	POS	CDV_Badger_Italy_90334_2026_PC	PZ871213	dead
**38**	dead	fox	-	Y A	08/05/2026	Val Tidone	-	-	-	tissue	POS	POS	CDV_fox_Italy_94580_2026_PC	PZ871214	dead
**39**	dead	badger	F	A	14/05/2026	Val Tidone	-	-	-	tissue	POS	POS	CDV_Badger_Italy_99270_2026_PC		dead
**40**	dead	fox	M	Y	22/05/2026	Val Tidone	-	-	-	tissue	POS	POS	CDV_fox_Italy_106844_2026_PC	PZ871211	dead
**41**	dead	fox	M	A	04/06/2026	Val Trebbia	-	-	-	tissue	NEG	NEG			dead
**42**	alive	fox	F	Y	04/06/2026	Val Trebbia	POS,16,1	-	-	tissue	POS	POS			dead
**43**	dead	badger	M	A	24/06/2026	Val Trebbia	-	-	-	tissue	NEG	NEG			dead

**CDV positivity in animals was defined by RT-PCR results on post mortem analysis. -: sample not available.*
